# Knock down of HIF-1α in glioma cells reduces migration *in vitro *and invasion *in vivo *and impairs their ability to form tumor spheres

**DOI:** 10.1186/1476-4598-9-133

**Published:** 2010-06-01

**Authors:** Olga Méndez, Jiri Zavadil, Mine Esencay, Yevgeniy Lukyanov, Daniel Santovasi, Shu-Chi Wang, Elizabeth W Newcomb, David Zagzag

**Affiliations:** 1 Microvascular and Molecular Neuro-oncology laboratory, New York University School of Medicine, New York University Langone Medical Center, New York, NY, USA; 2 Department of Pathology, New York University Langone Medical Center, New York, NY, USA; 3 Division of Neuropathology and Department of Neurosurgery, New York University School of Medicine, New York, NY, USA; 4 New York University Cancer Institute, New York, NY, USA; 5 New York University Center for Health Informatics and Bioinformatics, New York University Langone Medical Center, New York, NY, USA

## Abstract

**Background:**

Glioblastoma (GBM) is the most common and malignant primary intracranial human neoplasm. GBMs are characterized by the presence of extensive areas of necrosis and hypoxia. Hypoxia and its master regulator, hypoxia inducible factor 1 (HIF-1) play a key role in glioma invasion.

**Results:**

To further elucidate the functional role of HIF-1α in glioma cell migration *in vitro *and in invasion *in vivo*, we used a shRNA approach to knock down HIF-1α expression complemented with genome-wide expression profiling, performed in both normoxic and hypoxic conditions. Our data show that knock down of HIF-1α in glioma cells significantly impairs their migration *in vitro *as well as their ability to invade into the brain parenchyma *in vivo*. Next, we assessed the role that HIF-1α plays in maintaining the characteristics of cancer stem cells (CSCs). By using the tumor sphere forming assay, we demonstrate that HIF-1α plays a role in the survival and self-renewal potential of CSCs. Finally, expression profiling experiments in glioma cells provided detailed insight into a broad range of specific biological pathways and processes downstream of HIF-1α. We discuss the role of these processes in the migratory and invasive properties, as well as the stem cell biology of glioblastomas

**Conclusions:**

Our data show that knock down of HIF-1α in human and murine glioma cells impairs their migration *in vitro *and their invasion *in vivo*. In addition, our data suggest that HIF-1α plays a role in the survival and self-renewal potential of CSCs and identify genes that might further elucidate the role of HIF-1α in tumor migration, invasion and stem cell biology.

## Background

Glioblastoma (GBM) is the most common and malignant primary central nervous system tumor [1-3]. GBMs are characterized by a high degree of invasion, angiogenesis and the presence of necrosis [2,3]. In addition, these tumors are hypoxic [4-6]. Hypoxia and its master regulator hypoxia inducible factor 1 (HIF-1) play a key role in glioma invasion [4,6]. In GBMs, HIF-1α is primarily localized in pseudopalisading cells around areas of necrosis and in tumor cells infiltrating the brain at the invasive edge of the tumor [6]. Its expression appears to be associated with intratumoral hypoxia and correlates with glioma grade and vessel density, emphasizing its role in brain tumor progression and angiogenesis [6]. In addition to oxygen levels, HIF-1 expression can be affected by several mechanisms including the activation of oncogenes such as EGFR or loss of tumor suppressors, such as p53 or PTEN, both of which are common alterations found in GBMs [4].

HIF-1 is a heterodimeric transcription factor that consists of 2 subunits. The HIF-1β subunit is constitutively expressed whereas the HIF-1α subunit is regulated by oxygen levels. It is stable under hypoxic conditions but is rapidly degraded under normoxic conditions [7]. After stabilization or activation, HIF-1 translocates to the nucleus where it induces the transcription of numerous downstream target genes via their hypoxia response elements (HREs) [7]. One of the target genes is vascular endothelial growth factor (VEGF), an important angiogenic factor. HIF-1 acts as an activator of angiogenesis by controlling the expression of VEGF as well as other proangiogenic factors such as placenta-like growth factor and platelet-derived growth factor β [4,5].

Growing evidence suggests the existence of a reservoir of cells within the tumor that share similar properties with normal stem cells and are capable of driving tumorigenesis [8,9]. These cells, called cancer stem cells (CSCs) or tumor initiating cells, have been described in several tumor types including GBMs [9]. CSCs are cells with extensive proliferation, self-renewal and tumor initiation properties [10,11]. In addition, brain tumor stem cells have the ability to grow as nonadherent spheres when grown in the proper culture media [12]. It has been shown that hypoxia is able to maintain the undifferentiated state of stem cells [13]. Furthermore, it has been shown that hypoxia is able to promote the survival and proliferation of certain populations of neural stem cells or neural progenitor cells [14].

To further elucidate the role that HIF-1α has in glioma cell migration *in vitro *and *in vivo*, we knocked down the expression of HIF-1α and evaluated the migration and invasion potential of these glioma cells. In addition, we assessed the role that HIF-1α plays in maintaining CSCs. To identify genetic pathways that might be involved in the reduced migration *in vitro*, the reduced invasiveness *in vivo *and the reduced ability to form tumor spheres of cells knocked down for HIF-1α, we performed gene expression profiling analysis. Our data show that knock down of HIF-1α in human and murine glioma cells impairs their migration *in vitro *and their invasion into the brain parenchyma *in vivo*. In addition, our data suggest that HIF-1α plays a role in the survival and self-renewal potential of CSCs and identify genes that might further elucidate the role of HIF-1α in tumor migration, invasion and stem cell biology.

## Methods

### Cell culture

The human glioma cell lines, LN308 (kindly provided by Dr. F. Furnari from the Ludwig Institute for Cancer Research, UCSD) and U87MG, the murine glioma cell line GL261 tagged with green fluorescent protein (GFP) [15], and the human embryonic kidney (HEK) 293T cells, used for lentivirus production (kindly provided by Dr. M. Pagano, New York University) were cultured in 5% CO_2 _and 95% humidified atmosphere air at 37°C in Dulbecco's modified Eagle's medium (DMEM; Cellgro, Herndon, VA), supplemented with 10% fetal bovine serum (FBS; Atlanta Biologicals, Lawrenceville, GA), 1% penicillin and streptomycin, and 2 mM glutamine (Gibco; Grand Island, NY).

For hypoxic exposure, cells were placed in a sealed Modular Incubator Chamber (Billups-Rothenberg Inc., Del Mar, CA) flushed with 1% O_2_, 5% CO_2_, and 94% N_2 _and incubated at 37°C for 16 h.

### Western blot analysis

Cells (1 × 10^6^) were seeded in 10 cm^2 ^dishes in complete growth medium. The following day, cells were exposed to normoxic (20% O_2_) or hypoxic conditions (1% O_2_) for 16 h. Protein quantitation and electrophoresis was performed as previously described [16]. Western blot analysis was performed with the following antibodies: mouse anti-HIF-1α monoclonal antibody used at 1:1000 (clone 54, BD Transduction Laboratories, San Jose, CA), rabbit anti-HIF-1α used at 1:1000 (Cat. A300-286A Bethyl Laboratories, Inc, Montgomery, TX) and mouse anti-actin monoclonal antibody used at 1:20,000 (clone C4, Chemicon International, Inc., Temecula, CA). Sheep anti-mouse and donkey anti-rabbit IgG (Amersham Life Pharmacia Biotech, Piscataway, NJ) horseradish peroxidase-conjugated secondary antibodies were used at 1:2000. Immunodetection was followed by visualization and densitometry using Scion Image NIH Image software (National Institutes of Health, Bethesda, MD).

### Lentivirus production and infection of glioma cells

The shRNA directed against HIF-1α used to infect the LN308 cell line (kindly provided by Dr. L. Gardner from New York University) was (CCG GAG AGG TGG ATA TGT CTG GGC TCG AGC CCA GAC ATA TCC ACC TCT TTT TT) and the scramble sequence was (CCG GGG GTC TGT ATA GGT GGA GAC TCG AGT CTC CAC CTA TAC AGA CCC TTT TT). Five different shRNA sequences directed against HIF-1α were purchased from Open Biosystems (Huntsville, AL) and used to knockdown human and mouse HIF-1α expression in U87MG and GL261 glioma cell lines.

Recombinant lentiviruses were produced by cotransfecting HEK 293T cells with the lentivirus expression vector (pLKO.1 puro) and packaging plasmids (Δ8.9 and vsv-g) using Fugene 6 (Roche Diagnostics, Indianapolis, IN) as a transfection reagent. Infectious lentiviruses were collected at 24, 48 and 72 h after transfection and the pooled supernatants centrifuged to remove cell debris and filtered through a 0.45 μm filtration unit. GFP-tagged murine GL261 and human glioma cells were infected and stable transfectants were selected in puromycin for 7 days. After this time, cells were expanded and exposed to normoxic or hypoxic conditions for 16 h to test for HIF-1α downregulation. Two to three of the five shRNA sequences were able to efficiently downregulate HIF-1α expression in glioma cells based on Western blot analysis. These lentivirus expression vectors (shHIF) were used for further investigations.

### Boyden chamber migration assay

BD Biocoat chambers (BD Biosciences Discovery Labware, Bedford, MA) with 8-μm pore size polystyrene filter inserts for 24-well plates were used according to the manufacturer's instructions and as described [16]. Briefly, cells (2 × 10^4^) were seeded onto the upper compartment of each chamber in 300 μl of DMEM with 10% FBS and placed into wells containing 750 μl of complete medium in the lower chamber. The migration chambers were incubated for 16 h in normoxic or hypoxic conditions at 37°C. Following incubation, the inserts were fixed and stained and the number of migrating cells was counted as described [17]. Two independent experiments, done in duplicate, were performed. Images were quantified using Image-Pro Discovery software.

### Animal experiments

Female C57BL/6 mice (10-12 weeks old) were purchased from Taconic (Germantown, NY) and maintained in accordance with the protocol approved by the Institutional Animal Care and Use Committee. GFP-tagged GL261 stable transfectants (1 × 10^5^) expressing either the empty vector or the HIF-1α shRNA sequence were implanted stereotactically into mouse brains as previously described [18]. Animals were sacrificed when they became moribund and brains were removed and processed for paraffin embedding. Tumors in the hematoxylin and eosin (H&E) coronal sections were measured to determine tumor volumes and depth of invasion. Tumor volume was calculated using the formula: *L *× *S*^2 ^× 1/2, where *L *is the longest tumor diameter and *S *the shortest tumor diameter as described [19]. To assess depth of invasion, H&E and GFP stained slides were photographed at × 4 using a resolution of 432 dpi. Images were assembled using Adobe Photoshop CS3 Extended Software. A line was drawn along each evaluable surface, establishing a border of the tumor core without any invading cells or projections of groups of cells. A tumor with 0% of invasion would have a true tumor border falling directly on this line. Then, at an interval of 200 to 400 pixels along this line, measurements were taken from the previously drawn line to the furthest invading cell perpendicular to solid line away from the tumor border corresponding to the drawn line. Two independent experiments were performed. The total number of measurements for each tumor was analyzed for statistical significance. The number of measurements per animal averaged 64.

### Immunohistochemistry

To determine HIF-1α expression, sections were stained using the Catalyzed Signal Amplification System (DAKO, Carpinteria, CA). After deparaffinization and rehydration, slides were treated with target retrieval solution (DAKO) at 97°C for 45 min. Nuclei were counterstained with hematoxylin. Negative controls were done with nonimmune serum or PBS used instead of primary antibody. Primary antibody was used as follows: HIF-1α 1:300 (Bethyl Laboratories). Automated analysis was done on a Tech-Mate 100 automated stainer (Ventana-BioTek Solutions, Inc., Tucson, AZ).

### Tumor sphere forming assay

Cells were diluted in serum-free growth medium (1000 cells/ml) and plated in 100 μl in non-tissue culture coated 96-well plate. Cells were fed with 25 μl of serum-free growth medium every other day, for 14 days. The culture medium consisted of serum-free DMEM/F12 (Invitrogen, Grand Island, NY) supplemented with 10 U/ml heparin, 2% B27 (Invitrogen), human recombinant fibroblast growth factor 2 (FGF-2, 20 ng/ml, Sigma-Aldrich, St. Louis, MO) and epidermal growth factor (EGF, 20 ng/ml, Sigma-Aldrich). After 14 days, spheres were measured and those > 100 μm were counted as a tumor sphere forming unit. The data calculated for the number and size of the tumor spheres is the average of three independent experiments. Spheres were counted and measured from 32 different wells/cell line/experiment.

### cRNA preparation and microarray hybridization

Total RNA was extracted using Trizol (Invitrogen) as previously described [20], from GL261 control and GL261 shHIF cells after 16 h of incubation in either normoxic or hypoxic conditions. RNA quality and quantity was determined using Agilent 2100 Bioanalyzer and Nanodrop ND-1000. 5 μg of total RNA were used to prepare cRNA following the Affymetrix one-cycle labeling protocol and standardized array processing procedures recommended by Affymetrix (Santa Clara, CA), including hybridization, fluidics processing and scanning of the Affymetrix mouse Genechip MG-430A 2.0 arrays containing 22,000 probes (14,000 well-characterized mouse genes). Two independent experiments were performed. Duplicates of each studied condition (control *vs*. shHIF in normoxia *vs*. hypoxia) were processed for microarray analysis.

### Array data analysis

The raw data (Affymetrix CEL files) were normalized using Robust Multichip Average algorithm (RMA) [21] in GeneSpring GX software (Agilent Technologies, Santa Clara, CA). Genes/mRNAs differentially regulated between the studied conditions were identified by feature selection algorithm Pavlidis Template Matching (PTM, p < 0.05) incorporated in the Multi-experiment Viewer of the TIGR TM4 Analysis package [22]. Gene lists resulting from the statistical analysis results were further thresholded based on fold difference in expression (> 30%). The GO (Gene Ontology) and KEGG (Kyoto Encyclopedia of Genes and Genomes) pathway functional annotations were performed using the web-based tool DAVID bioinformatics resources [23].

### Statistical methodologies

Statistical significance was determined by unpaired t-test (GraphPad Prism Software).

## Results

### Knock down of HIF-1α reduces hypoxia-induced HIF-1α expression in glioma cells

**Figure 1:**
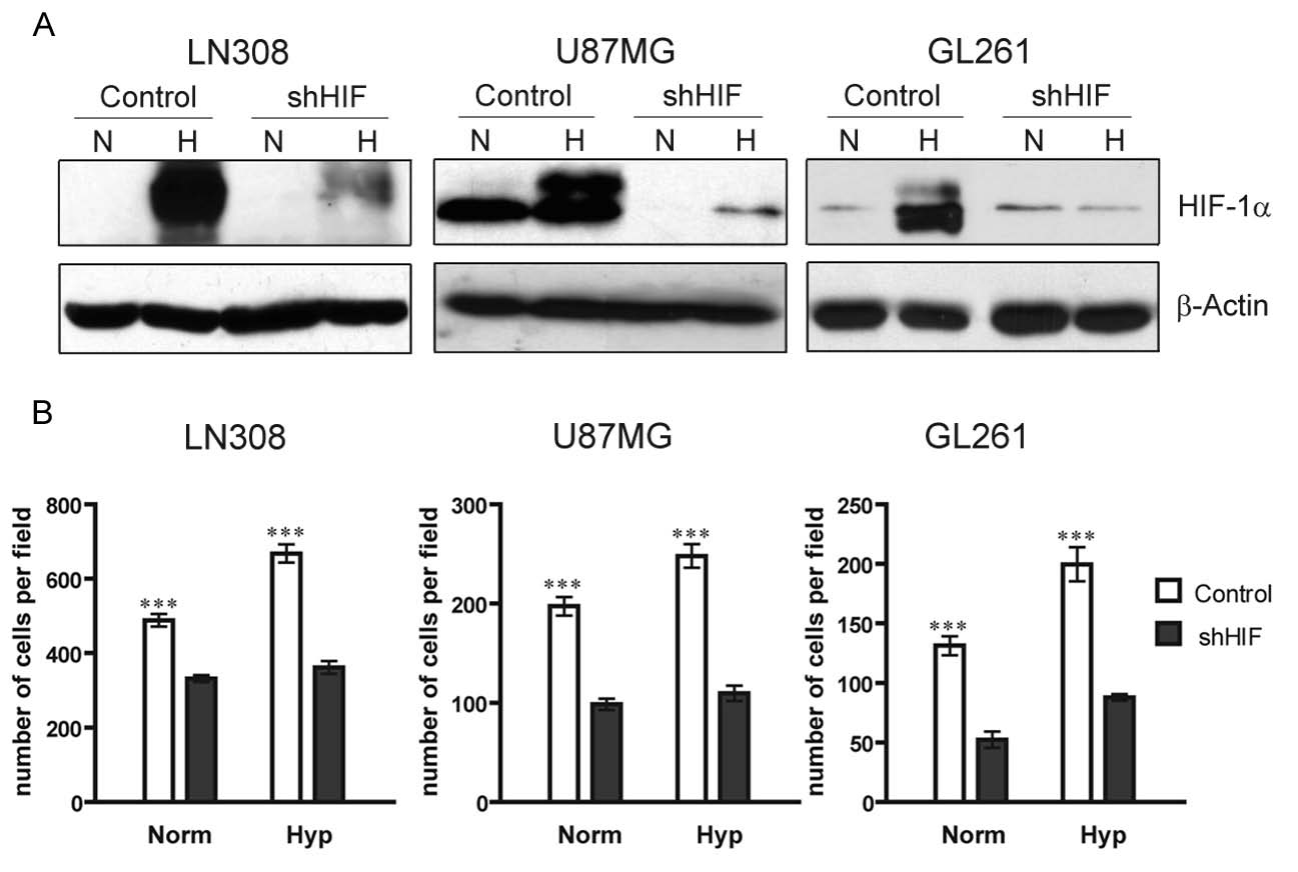
**Knock down of HIF-1α reduces hypoxia-induced HIF-1α expression and hypoxia-induced migration of glioma cells**. (**A**) Western blot showing the expression level of HIF-1α under normoxic (Norm) and hypoxic (Hyp) conditions in human (LN308 and U87MG), and murine (GL261) glioma cell lines, after HIF-1α shRNA lentivirus-mediated infection (shHIF). β-Actin was used as loading control. A representative result of two independent experiments is shown. (**B**) Knock down of HIF-1α reduces hypoxia-induced migration of glioma cells. Boyden chamber assays showing migration of human (LN308 and U87MG) and murine (GL261) glioma cell lines infected with HIF-1 shRNA (shHIF) or the control cells. Two independent experiments, done in duplicate, were performed. Each graph represents the number of cells per field counted after 16 h of migration in either a normoxic (Norm) or a hypoxic (Hyp) environment (three asterisks, p < 0.0001).

In order to understand the role that HIF-1α plays in glioma cell invasion, we used a shRNA approach to knock down its expression. We studied how hypoxia, a common feature of human GBMs, can affect this behavior *in vitro*. To accomplish this, we used stable transfectants (shHIF and control) of both human (LN308 and U87MG) and murine (GL261) glioma cell lines and compared the levels of expression of HIF-1α under both normoxic and hypoxic conditions (Figure 1A).

Under hypoxic conditions, all glioma cell lines used as a control showed induction of HIF-1α. HIF-1α levels of expression were increased in LN308, U87MG and GL261 cells by 9-, 2- and 14-fold, respectively. We detected two HIF-1α bands under hypoxic conditions. It has been shown that HIF-1α is expressed as two bands that represent post-translational modifications [24]. In contrast, when HIF-1α expression was knocked down in human and murine glioma cells the levels of HIF-1α were lower in normoxic conditions and failed to become upregulated in hypoxic conditions (Figure 1A).

### Knock down of HIF-1α reduces hypoxia-induced migration of glioma cells in the Boyden chamber assay

To study the role of HIF-1α and hypoxia in glioma cell migration we analyzed the ability of stable transfectants expressing shHIF to migrate under both normoxic and hypoxic conditions using the Boyden chamber assay (Figure 1B). In hypoxic conditions, all cell lines increased their migration significantly compared to control cultures in normoxic conditions (p < 0.001). In all cases, the control glioma cells showed significantly higher numbers of migrated cells under normoxic conditions compared with cells knocked down for HIF-1α expression (p < 0.0001). Only murine GL261 cells knocked down for HIF-1α expression showed a significant increase in migration in hypoxic conditions compared to similar cultures in normoxic conditions (p < 0.001). Nevertheless, under both normoxic and hypoxic conditions, knock down of HIF-1α significantly reduced the number of migrated cells in all cell lines (p < 0.0001).

### Knock down of HIF-1α reduces invasion of murine glioma cells in vivo

GL261 cells infected either with the empty vector or a HIF-1α shRNA were injected into the brains of C57BL/6 mice. Animals were sacrificed when they became moribund. Brains were harvested and processed for histopathology and immunohistochemistry.

There was no significant difference in the average survival between the two groups. Animals injected with control cells survived 24.1 ± 1.8 days compared with 26.0 ± 1.7 days for animals injected with shHIF cells. Consistent with survival outcome, tumor volumes showed no significant differences between the two groups. Tumors derived from cells infected with the empty vector had volumes of 64.9 ± 17.5 mm^3 ^compared with volumes of 77.1 ± 12.5 mm^3 ^in tumors derived from cells knocked down for HIF-1α.

**Figure 2:**
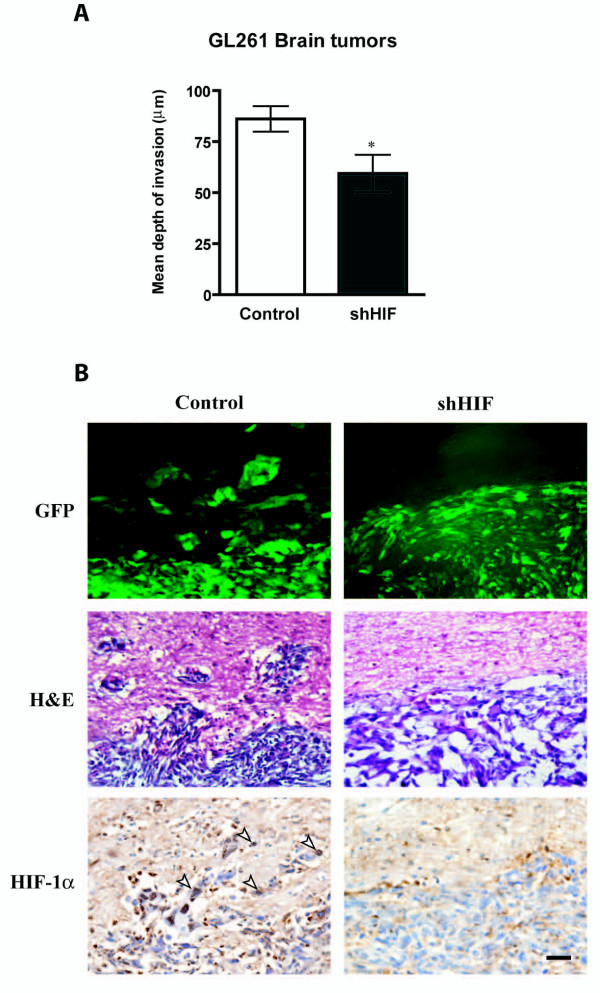
**Knock down of HIF-1α reduces invasion of murine glioma cells *in vivo*.** Representative tumors derived from GFP-tagged GL261 shHIF-1 and control cells showing tumor sections stained for GFP, H&E and HIF-1α. (**A) **The bar graph represents the average depth of invasion (μm) at the edge of tumors derived from GL261 control and GL261 shHIF cells (N = 7/group) (asterisk denotes p = 0.03). (**B**) As shown by GFP staining and confirmed by H&E, control tumors derived from cells expressing HIF-1α displayed an invasive edge compared with tumors derived from GL261 shHIF-1 cells with marginal invasion. HIF-1α immunohistochemistry confirms lack of HIF-1 expression in HIF-1α knockdown tumors and its expression in the controls. Open arrowheads point to HIF-1α expressing cells at the infiltrative margin in the control tumors. Scale bar represents 50 μm.

Importantly, tumors derived from cells lacking HIF-1α expression were significantly less invasive compared with control tumors expressing HIF-1α, as assessed by both immunofluorescence for GFP and H&E staining (Figure 2). The mean depth of invasion in control tumors was 86.1 ± 6.2 μm compared with 59.4 ± 9.1 μm in tumors derived from cells knocked down for HIF-1α (p = 0.03).

Immunostaining of tumor sections for HIF-1α expression confirmed that the tumors resulting from the injection of shHIF cells had decreased HIF-1α expression compared with control tumors resulting from the injection of cells infected with the empty vector (Figure 2B).

### Knock down of HIF-1α reduces the ability of murine glioma cells to form tumor spheres

**Figure 3:**
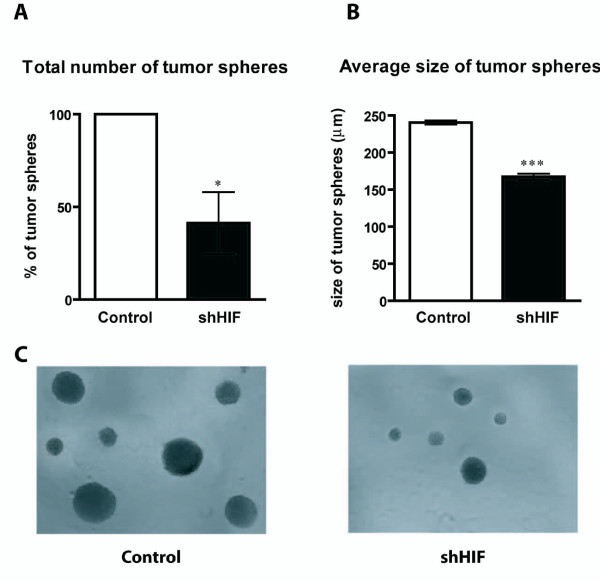
**Knock down of HIF-1α reduces the ability of murine glioma cells to form tumor spheres**. Primary tumor sphere forming capacity of GL261 shHIF cells in comparison with control cells. Three independent experiments were performed. **(A) **Bar graph represents the % of the total number of tumor spheres in shHIF cells *versus *the control. Knockdown of HIF-1α significantly reduces the ability of glioma cells to form tumor spheres. The shHIF cells formed 60% fewer tumor spheres compared with control cells (asterisk denotes p = 0.02). (**B**) Bar graph represents the average diameter (μm) of tumor spheres in shHIF cells *versus *the control. Lack of HIF-1α significantly reduces the average diameter of the spheres formed (167.4 ± 4.1 μm) compared with the size of those derived from control cells (240.6 ± 2.8 μm) (three asterisks denotes p < 0.0001). (**C**) Representative images of tumor spheres derived from either control or shHIF cells.

It has been shown that when grown in anchorage-independent and serum-free conditions, glioma cells are able to form tumor spheres. These tumor-derived spheres are known to be enriched in CSCs [25]. Cells infected with the empty vector or knocked down for HIF-1α were plated in the tumor sphere forming assay and the number and size of the tumor spheres were analyzed on day 14. Cells knocked down for HIF-1α formed significantly fewer tumor spheres than control cells. The shHIF cells formed 60% fewer tumor spheres compared with the number of tumor spheres formed by the control cells (p = 0.02) (Figure 3A). In addition, the size of the tumor spheres was also significantly reduced in cells knocked down for HIF-1α. The average diameter of the spheres formed by control cells was 240.6 ± 2.8 μm compared with 167.4 ± 4.1 μm for the spheres formed by shHIF cells (p < 0.0001) (Figure 3B).

### Knock down of HIF-1α alters the gene expression profile induced by hypoxia

To identify genetic pathways in shHIF cells that might be involved in the reduced migration *in vitro*, the reduced invasiveness *in vivo *and the reduced ability to form tumor spheres, we performed gene expression profiling using Affymetrix oligonucleotide arrays. Cells infected with the empty vector or knocked down for HIF-1α were grown under normoxic or hypoxic conditions for 16 h. Two independent experiments were performed and subjected to statistical analysis that identified reproducibly modulated genes correlating and potentially functionally associated with the altered characteristics identified in shHIF cells.

**Figure 4:**
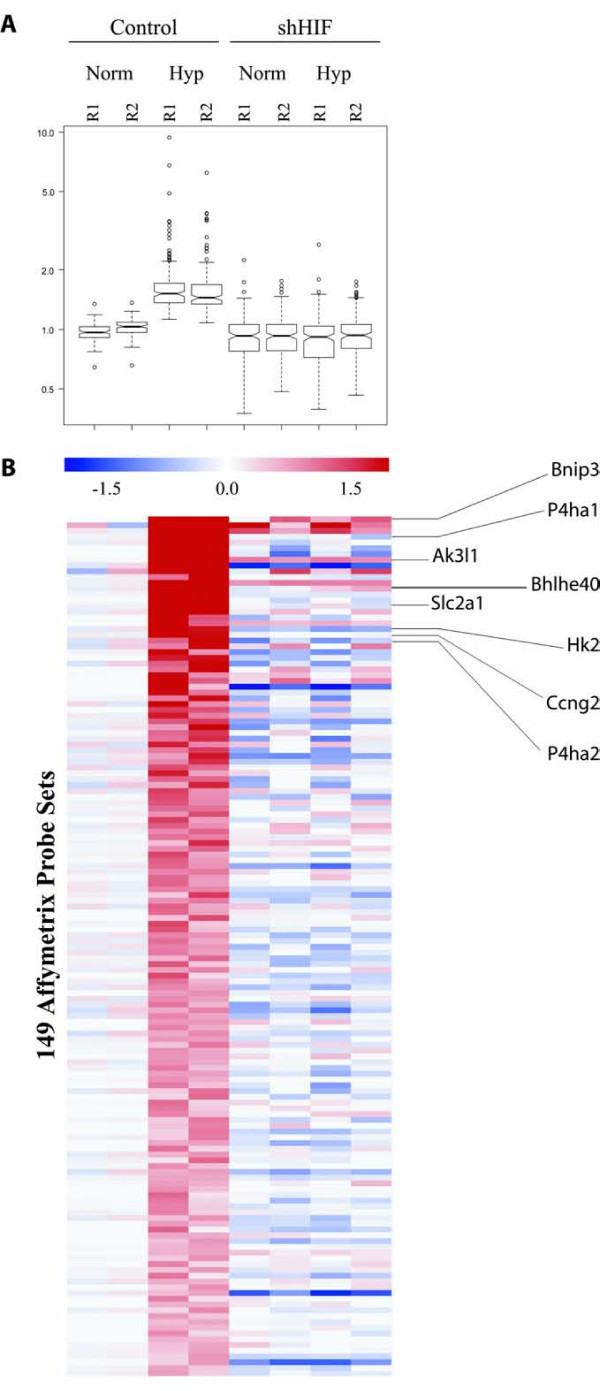
**Profile of genes that are upregulated in response to hypoxia in GL261 control cells and show no change or downregulation in GL261 shHIF cells, using as normalization baseline the GL261 control cells in normoxia**. For each cell line (control and shHIF) and condition: Norm (Normoxia) and Hyp (Hypoxia) we show the results from two independent experiments used in the analysis (R1 and R2). (**A**) Box plot analysis shows a five-number summary (the smallest observation, lower quartile, median, upper quartile, and largest observation) for the 149 significantly modulated Affymetrix Probe sets, found by Pavlidis Template Matching (p < 0.05), based on two individual experiments (R1 and R2). (**B**) Heat map showing the 149 Affymetrix probe sets including known HIF-1α targets that are upregulated in response to hypoxia in GL261 control cells and show no change or downregulation in GL261 shHIF cells. Red and blue colors denote increased and decreased mRNA abundance, respectively.

The analysis of the genes that were upregulated in the control cells in response to hypoxia showed a number of known HIF-1α target genes such as Bnip3, P4ha1, Ak3l1, Bhlhe40, Slc2a1 (GLUT1), Vegfa, Hk2, Ccng2 and P4ha2 (Table 1). In contrast, when shHIF cells were exposed to hypoxia, the same genes failed to become upregulated, validating our shRNA approach to block HIF-1α expression, and the response of shHIF cells to hypoxia. When we analyzed the genes that were reproducibly upregulated in response to hypoxia in the control cells but showed neither increase nor downregulation in shHIF cells, using as normalization baseline the GL261 control cells in normoxia, we identified 149 Affymetrix probe sets (Figure 4). Using this normalization, the levels of Vegfa, a well known HIF-1α target, showed a 2.2 fold increase in response to hypoxia in GL261 control cells. In contrast, Vegfa levels in shHIF cells did not show a hypoxic upregulation (Additional file 1, Figure S1). Although HIF-1α has been recognized as a potent stimulator of VEGF expression, there are other molecules that control VEGF expression by positive or negative feedback loops. In Figure 4, we show the Box plot analysis (Figure 4A) and the heat map (Figure 4B) of the probe sets that follow this distinct profile with the statistical significance described in Material and Methods. The complete and fully annotated set of 149 genes identified by this analysis is shown in Additional file 2, Table S1.

**Table 1:** HIF-1α target genes upregulated in response to hypoxia in GL261 control cells

Affymetrix ID	Gene Symbol	Gene Title	Fold Difference	SD
1422470_at	**Bnip3**	BCL2/adenovirus E1B 19kDa-interacting protein 1, NIP3	7.83	± 2.26
1452094_at	**P4ha1**	Procollagen-proline, 2-oxoglutarate 4-dioxygenase (proline 4-hydroxylase), alpha 1 polypeptide	3.46	± 0.60
1421830_at	**Ak3l1**	Adenylate kinase 3-like 1	3.09	± 0.58
1418025_at	**Bhlhe40**	Basic helix-loop-helix family, member e40	2.54	± 0.04
1426599_a_at	**Slc2a1**	Solute carrier family 2 (facilitated glucose transporter), member 1	2.24	± 0.03
1420909_at	**Vegfa**	Vascular endothelial growth factor A	2.19	± 0.48
1422612_at	**Hk2**	Hexokinase 2	2.08	± 0.20
1416488_at	**Ccng2**	Cyclin G2	2.05	± 0.60
1417149_at	**P4ha2**	Procollagen-proline, 2-oxoglutarate 4-dioxygenase (proline 4-hydroxylase), alpha II polypeptide	2.05	± 0.12

List of HIF-1α target genes found in the gene expression profile experiment as upregulated in response to hypoxia in GL261 control cells. For each gene the Affymetrix ID, the gene symbol, gene title, average fold difference and standard deviation (n = 2) are shown.

### Genes differentially expressed in response to hypoxia in shHIF cells versus control cells

Next, we analyzed those genes that showed differential expression under hypoxic conditions, either up- or downregulated, in the shHIF cells compared with the control cells (Table 2). The table shows the fold differences for genes that are differentially expressed between shHIF and control cells in hypoxic conditions. Some genes were upregulated in response to hypoxia in shHIF cells despite the absence of HIF-1α expression. This gene set included Thrombospondin 1 and the member of the DNA damage repair response effectors H2afx (Table 2). Among the genes that were downregulated in shHIF cells in response to hypoxia were Adamts-5, MAP4K4, Jmjd1 and Ceruloplasmin. Reduced levels of the metalloprotease Adamts-5 and the mitogen-activated protein kinase MAP4K4 might contribute to the less invasive nature of shHIF tumors. Downregulation of Jmjd1 and Ceruloplasmin, genes associated with stem cells, might be correlated with the poor ability of shHIF cells to grow as tumor spheres.

**Table 2:** Genes differentially expressed in response to hypoxia in shHIF cells *versus *control cells

Affymetrix ID	Gene Symbol	Gene Title	Fold Difference	SD
1460302_at	**Thbs1**	Thrombospondin 1	11.76	± 2.27
1416746_at	**H2afx**	H2A histone family, member X	5.23	± 0.19
1422561_at	**Adamts5**	A disintegrin-like and metalloprotease (reprolysin type) with thrombospondin type 1 motif, 5 (aggrecanase-2)	0.49	± 0.03
1448050_s_at	**Map4k4**	Mitogen-activated protein kinase kinase kinase kinase 4	0.33	± 0.02
1417495_×_at	**Cp**	Ceruloplasmin	0.25	± 0.01
1426810_at	**Jmjd1**	Jumonji domain containing 1	0.15	± 0.02

The fold differences for genes that are differentially expressed between shHIF and control cells in hypoxic conditions. For each gene the Affymetrix ID, the gene symbol, gene title, average fold difference and standard deviation (n = 2) are shown

### Pathway analysis of HIF-1α-dependent genetic programs

The GO and KEGG pathway functional annotations of genes differentially expressed in response to hypoxia in shHIF cells functionally grouped genes predominantly involved in DNA repair (Rad50, Rad21, Rad52, Trp53bp1), cell adhesion (Col17a1, Twist1) and cell migration (Ephb3, Kitl, Erbb2) among others. We also detected several genes of the Wnt (Fzd2, Fzd6, Tcf7l2) and the TGF-β pathway (Smad4, Smad2, Map3K7) (Additional file 3, Table S2). Taken together, the alteration of expression of these genes and pathways might be associated with the decreased migration and invasion potential of shHIF cells.

## Discussion

This study shows that knock down of HIF-1α in glioma cells results in i) reduced migration *in vitro*, ii) decreased invasion *in vivo*, iii) reduced ability to form tumor spheres and iv) altered gene expression profiles under hypoxic conditions. The results identified genes that might contribute to glioma cell invasion induced by hypoxia.

Hypoxia is a critical aspect of the glioma microenvironment, and it has been associated with poor prognosis, increased angiogenesis, tumor growth and resistance to radio- and chemotherapy [26]. HIF-1α, the key hypoxia regulatory factor, has been shown to be important in promoting both angiogenesis and invasion [4]. The role that hypoxia plays in glioma cell migration is well established [17,27,28]. Our *in vitro *data show that hypoxia increases migration of human and murine glioma cells. The shRNA approach directed towards HIF-1α resulted in reduced migration of human and murine glioma cells knocked down for HIF-1α compared with control cells with normal HIF-1α expression. These data support the role of HIF-1α and hypoxia in glioma cell migration.

Our data did not show any significant differences in overall survival or tumor volume between animals injected with cells knocked down for HIF-1α expression or control cells. Some studies showed that reduction of HIF-1α levels by siRNA in glioma cells grown in mouse flanks decreased tumor growth which was associated with reduction of VEGF and GLUT-1, two known downstream targets of HIF-1α [29]. In another study, HIF-1α deficient teratomas grew faster, despite reduced vascularization, due to reduced apoptosis [30]. A similar result, showing reduced vascularization and accelerated tumor growth, was obtained with glioma cells injected subcutaneously, and expressing a HIF dominant negative construct [31]. HIF-1α is commonly expressed in tumors, though the role that HIF-1α deficiency plays in tumor growth is not fully understood.

It is important to consider the role of the interaction between the tumor cells and the host brain microenvironment on the invasion ability of tumor cells. Our data show that inhibition of HIF-1α by expression of a stable shRNA alters the growth pattern of glioma cells in the brain microenvironment. Tumors with lower HIF-1α expression are less invasive, as seen by a better defined border, while tumors derived from control cells demonstrated an irregular infiltrating edge. In concordance with our results, glioma cells knocked down for HIF-1α using an siRNA approach failed to invade the surrounding brain tissue, as assessed using an organotypic brain slice model [28].

There is growing evidence that in GBMs, as in other solid tumors, a small fraction of cells that have tumor initiating properties are present. These cells are defined as CSCs. GL261 glioma cells form neurospheres when grown in a serum free medium supplemented with FGF and EGF. Neurospheres are enriched in CSCs [25]. One study showed that neurospheres derived from GL261 cells resulted in more infiltrative and angiogenic tumors when implanted into the brains of mice [32]. Our data show that when HIF-1α expression is knocked down, the ability of GL261 cells to form tumor spheres is significantly reduced compared with the control cells. Although the reduced number and size of tumor spheres did not inhibit tumor growth *in vivo*, it correlated with reduced invasion of glioma cells into the brain parenchyma. Cancer stem cells are believed to persist in tumors and cause tumor recurrence and invasion, rendering conventional therapies ineffective. Therefore, a reduction in the number of tumor spheres might have important implications for decreasing the invasive potential of glioma cells. Recently Zhao *et al*. showed that knock down of HIF-1α was able to block the hypoxia induced migration of neural stem cells. This decrease in migration was due to reduced levels of HIF-1α and its downstream targets, such as CXCR4 [33]. Additionally, Li *et al*. recently showed that knock down of HIF-1α or HIF-2α in glioma stem cells impaired tumor sphere formation [34]. Hypoxia was reported to lead to an enrichment in neural stem cell markers [35], and reduction of HIF-1α levels abrogated this effect [36]. These studies, together with our findings, reinforce the importance of hypoxia and HIF-1α in cancer stem cell biology. Our data also suggest a relationship between a reduction in the number of tumor spheres and decreased invasiveness of glioma cells.

The hypoxic response driven by HIF-1α regulates the expression of genes involved in angiogenesis, invasion and epithelial-mesenchymal transition, indicating its role in local invasion [37,38]. The gene expression analysis identified genes and pathways that might further elucidate the role of HIF-1α in glioma invasion. Our analysis shows that when GL261 control cells were exposed to hypoxia, known HIF-1α targets such as Bnip3, P4ha1, Ak3l1, Bhlhe40, Slc2a1, Vegfa, HK2, Ccng2 and P4ha2 were upregulated. In contrast, these genes failed to become upregulated in cells knocked down for HIF-1α after hypoxia exposure.

Two of the genes identified through gene expression analysis that were downregulated in shHIF hypoxic cells, Adamts-5 and MAP4K4, might contribute to the less invasive growth of the GL261 tumors that we observed. Adamts-5 is a desintegrin and metalloproteinase with thrombospondin motifs that is able to degrade brevican, one of the components of the brain extracellular matrix. It has been shown that Adamts-5 is overexpressed in glioma cells compared with normal brain tissue and is involved in glioma invasion [39]. Degradation of brevican may facilitate the invasion of glioma cells to the surrounding brain tissue [40]. MAP4K4 is a serine/threonine kinase commonly overexpressed in tumors. Its role in tumor progression was demonstrated using siRNA. Knock down of MAP4K4 resulted in inhibition of tumor cell migration and invasion [41], [42]. In contrast, overexpression of MAP4K4 increased invasion in the presence of hepatocyte growth factor [43].

Some of the genes upregulated in response to hypoxia in shHIF cells were Thrombospondin 1 and H2afx that might contribute to reduced migration *in vitro *and invasion *in vivo*. Thrombospondin 1 is a multifunctional extracellular matrix protein that functions as an angiogenic inhibitor and is involved in activating latent TGF-β secreted by tumor cells [44]. Thrombospondin 1 inversely correlates with invasiveness and lymph node metastasis, and in lung adenocarcinoma cells it has been shown to directly inhibit invasion [45]. In endothelial cells, its overexpression reduces their migration ability *in vitro *[46]. H2afx, a member of the DNA damage repair response effectors is needed for endothelial cells to maintain their proliferation under hypoxic conditions and is crucial for hypoxia-driven neovascularization [47].

Two of the genes that might be associated with the poor ability of shHIF cells to grow as tumor spheres are Jmjd1 and Ceruloplasmin. Both genes are downregulated in shHIF cells exposed to hypoxic conditions. Jmjd1, a known HIF-1α target, is a histone demethylase positively regulated by the ES cell transcription factor Oct4. Jmjd1 has been shown to be critical in the maintenance of pluripotency of stem cells [48]. Ceruloplasmin is a copper protein inducible by hypoxia. Its expression has been shown to be enriched in highly malignant glioma stem-like cells [49].

## Conclusions

Taken together, our *in vitro *and *in vivo *data confirm the functional importance of HIF-1α in the invasive ability of GBM cells. Glioma cells with reduced HIF-1α expression and their response to hypoxia results in cells with reduced migration ability, overall less invasive tumors, and reduced ability to form tumor spheres. These observations, together with the gene expression analysis data, highlight the role of HIF-1α in glioma invasion and stem cell biology and identify genes that might further elucidate its role in tumor initiation and invasiveness.

## Abbreviations

GBM: (Glioblastoma); (HIF-1): hypoxia inducible factor 1; (CSCs): cancer stem cells; shHIF: (cells expressing an shRNA sequence directed to HIF-1α).

## Competing interests

The authors declare that they have no competing interests.

## Authors' contributions

OM designed experiments, performed shRNA knockdown, migration assays, array analysis, western blot and tumor spheres quantification and drafted the manuscript. JZ did array analysis and critically revised the paper. ME performed tumor sphere and differentiation experiments. YL did the animal experiments, SYW did the tumor sphere experiment, DS did the measurements of *in vivo *invasion. EWN and DZ conceived the study and critically revised the manuscript. All authors read and approved the final version of the manuscript.

## Supplementary Material

Additional file 1 **Figure S1**. Vegfa levels using as normalization baseline the values of GL261 control cells in normoxia. The graph show an increase of Vegfa levels in response to hypoxia in GL261 control cells, and no change in cells knockdown for HIF-1α. Asterisks denote statistical significance, as determined by two-tailed *t *test. One asterisk, p < 0.05, and two asterisks, p < 0.01. Click here for file

Additional file 2 **Table S1**. List of 149 genes upregulated in the control cells in response to hypoxia that showed no change or downregulation in shHIF cells, when using as normalization baseline the GL261 control cells in normoxia. Click here for file

Additional file 3 **Table S2**. Functional analysis of genes differentially expressed in shHIF cells in response to hypoxia as compared with control cells. The table lists GO and KEGG Pathway categories significantly enriched for specific gene effectors with candidate roles for the reduce migration and reduced invasion of glioma cells knocked down for HIF-1α. Yellow and blue colors, denote increase and decreased mRNA abundance, respectively. Click here for file
