# Supplementary material for: Knock down of HIF-1α in glioma cells reduces migration in vitro and invasion in vivo and impairs their ability to form tumor spheres
Source: Mol Cancer. 2010 Jun 1;9:133. doi: 10.1186/1476-4598-9-133 (PMC2896954; doi:10.1186/1476-4598-9-133)
Supplement: Additional file 1 — Figure S1. Vegfa levels using as normalization baseline the values of GL261 control cells in normoxia. The graph show an increase of Vegfa levels in response to hypoxia in GL261 control cells, and no change in cells knockdown for HIF-1α. Asterisks denote statistical significance, as determined by two-tailed t test. One asterisk, p < 0.05, and two asterisks, p < 0.01. [file 1476-4598-9-133-S1.DOC]

🞵🞵

🞵

🞵

**Additional Figure 1 – Figure S1**
